# 

**Published:** 1864-03

**Authors:** 


### CIRCULAR.

SURGEON-GENERAL'S OFFICE,
RICHMOND, Oct. 31, 1863.

The following Prospectus meets with the hearty approval of the Surgeon General, and the medical officers of the Confederate States service are earnestly requested to co-operate in the undertaking, and to forward their names, enclosing subscriptions, with as little delay as practicable.

S. P. MOORE, *Surgeon-General.*

CONFEDERATE STATES

MEDICAL AND SURGICAL JOURNAL,

Published under the auspices of the Surgeon-General.

**PRICE, $10 A YEAR IN ADVANCE.**

The CONFEDERATE STATES MEDICAL AND SURGICAL JOURNAL will be published monthly, in quarto form, and issued regularly to subscribers, who have paid in advance, at TEN DOLLARS A YEAR.

Communications intended for insertion will be addressed to Editor of "Confederate States Medical and Surgical Journal," care of Ayres & Wade, Richmond.

All persons who favor the enterprise, whether in or out of the army, are requested to act as agents. Subscription and other letters upon business should be addressed to AYRES & WADE,
Publishers and Proprietors,
Richmond, Va.

